# Evaluation of deep learning-based autosegmentation in breast cancer radiotherapy

**DOI:** 10.1186/s13014-021-01923-1

**Published:** 2021-10-14

**Authors:** Hwa Kyung Byun, Jee Suk Chang, Min Seo Choi, Jaehee Chun, Jinhong Jung, Chiyoung Jeong, Jin Sung Kim, Yongjin Chang, Seung Yeun Chung, Seungryul Lee, Yong Bae Kim

**Affiliations:** 1 Department of Radiation Oncology, Yonsei Cancer Center, Yonsei University College of Medicine, 50-1 Yonsei-ro, Seodaemun-gu, Seoul, 03722 South Korea; 2 Department of Radiation Oncology, Asan Medical Center, University of Ulsan College of Medicine, Seoul, 05505 South Korea; 3 Coreline Soft, Co., Ltd, Seoul, South Korea; 4 Department of Radiation Oncology, Ajou University School of Medicine, Suwon, South Korea; 5 Yonsei University College of Medicine, Seoul, South Korea

**Keywords:** Autocontouring, Breast, Organs at risk, Radiotherapy

## Abstract

**Purpose:**

To study the performance of a proposed deep learning-based autocontouring system in delineating organs at risk (OARs) in breast radiotherapy with a group of experts.

**Methods:**

Eleven experts from two institutions delineated nine OARs in 10 cases of adjuvant radiotherapy after breast-conserving surgery. Autocontours were then provided to the experts for correction. Overall, 110 manual contours, 110 corrected autocontours, and 10 autocontours of each type of OAR were analyzed. The Dice similarity coefficient (DSC) and Hausdorff distance (HD) were used to compare the degree of agreement between the best manual contour (chosen by an independent expert committee) and each autocontour, corrected autocontour, and manual contour. Higher DSCs and lower HDs indicated a better geometric overlap. The amount of time reduction using the autocontouring system was examined. User satisfaction was evaluated using a survey.

**Results:**

Manual contours, corrected autocontours, and autocontours had a similar accuracy in the average DSC value (0.88 vs. 0.90 vs. 0.90). The accuracy of autocontours ranked the second place, based on DSCs, and the first place, based on HDs among the manual contours. Interphysician variations among the experts were reduced in corrected autocontours, compared to variations in manual contours (DSC: 0.89–0.90 vs. 0.87–0.90; HD: 4.3–5.8 mm vs. 5.3–7.6 mm). Among the manual delineations, the breast contours had the largest variations, which improved most significantly with the autocontouring system. The total mean times for nine OARs were 37 min for manual contours and 6 min for corrected autocontours. The results of the survey revealed good user satisfaction.

**Conclusions:**

The autocontouring system had a similar performance in OARs as that of the experts’ manual contouring. This system can be valuable in improving the quality of breast radiotherapy and reducing interphysician variability in clinical practice.

**Supplementary Information:**

The online version contains supplementary material available at 10.1186/s13014-021-01923-1.

## Background

In breast cancer radiotherapy, techniques have evolved from two-dimensional (2D) radiotherapy planning to conformal radiotherapy planning and intensity-modulated radiation therapy [1]. Delineating and sparing organs at risk (OARs) has accordingly received attention recently in breast radiotherapy. In addition, three-dimensional (3D) computed tomography (CT)-based planning, which allows for the accurate assessment of each OAR receiving a radiation dose, has been increasingly used in modern radiotherapy. However, the delineation of OARs is a time-consuming and labor-intensive process and is prone to observer subjectivity, which results in interphysician variations.

With recent advances in big data collection and computing power, deep learning algorithms and procedures have increasingly been used in many different fields [2]. Medical image semantic segmentation, which relies on deep convolutional neural networks, has been extensively studied [3]. Unlike other image segmentation used in surgical and radiologic fields, normal tissue contouring in radiation oncology, known as “OAR delineation,” has been defined and standardized through expert consensus with regard to better quantification of dose-volume histogram–toxicity relationships [4]. Men et al. [5] previously developed deep learning-based target volume for breast radiotherapy, while Feng et al. [6] developed deep learning-based segmentation of OARs for thoracic radiotherapy. Our group also previously demonstrated the potential of deep learning-based autosegmentation of target volumes and OARs in breast cancer radiotherapy [7, 8]. A training set for a proposed deep learning-based autocontouring system (ACS) is generally generated by a single expert or a small group of experts [9]. Therefore, generalization is often discussed as an issue of external validity.

For application of the ACS in real-world clinical practice, its validation with experts from diverse clinical backgrounds, in terms of accuracy, time saving, and user satisfaction, would be necessary. However, our previous studies did not focus on the generalizability or real-world use [7, 8]. In this study, we evaluated the performance of a proposed ACS in delineating OARs for breast radiotherapy with a group of experts from multiple institutions.

## Methods

### ACS development

Training methods for deep neural networks have been described previously [7]. A home-made ACS was developed. Briefly, a single expert contoured the target volumes and OARs of 111 breast cancer patients who had received adjuvant radiotherapy after breast-conserving surgery. A three-dimensional (3D) U-Net-like convolutional neural network (CNN) was used, which was based on the U-Net structure [10], and combined with 3D version of EfficientNet-B0 as the backbone. Among the 111 cases, 92 were used as the training dataset, and 19 were used as the test dataset. Quantitative tests included the Dice similarity coefficient (DSC) and 95% Hausdorff distance (HD) and revealed an acceptable correlation between the autosegmented and manual contours. Qualitative tests included the scoring, after other panels reviewed the autocontours, and revealed acceptable results.

### Study design

There were 11 experts with a median of 7 years (range 2–21 years) of experience in breast cancer radiotherapy who volunteered to participate in this study. The experts were attending physicians (n = 2), clinical fellows (n = 6), residents (n = 2), and a dosimetrist (n = 1) from two institutions (Yonsei Cancer Center [Seoul, South Korea] and Asan Medical Center [Seoul, South Korea]). First, the 11 experts manually delineated the OARs (thyroid, right/left lung, spinal cord, esophagus, heart, liver, and right/left breast) in breast cancer radiotherapy on simulation CT scans of 10 women planning to undergo radiotherapy for breast cancer (i.e., manual contours). Second, an ACS was used on the same simulation CT scans, and these autocontours were provided to the experts. The experts were asked to correct the autocontours, as needed (i.e., corrected autocontours). Before contouring, CT scans were de-identified and the patients’ clinical information was blinded. The clinical treatment contours used for the patient’s radiotherapy delivery were removed to avoid bias during contouring. The experts were asked to record a video during contouring for each CT scan by using screen-recording software (oCam; OHSOFT, South Korea).

The best manual contours for each simulation CT were then selected as the ground truth, after a blind review of contour images of all CT slices by an independent third-party committee. The committee comprised five attending physicians in the radiation oncology department who were breast cancer authorities, and no member was part of the delineation group. Each member scored the performance of each contour, and ground truth was determined by the highest sum of scores. The second-best manual contours were determined by the second highest sum of scores. This blind review of contours was conducted online by using a questionnaire platform by Google (Menlo Park, CA). By using these ground truths, accuracy was compared between the manual autocontour, corrected autocontour, and autocontour groups.

### Endpoints

Endpoints were determined based on three aspects: (1) accuracy of OARs volumes and interphysician variability, (2) time-saving effect, and (3) user satisfaction. To assess accuracy of OARs volumes and interphysician variability, the DSC and 3D HD were used; a higher DSC and a lower HD indicated better geometric overlap. DSC was defined as *D*(*A,B*) = *2|A* ∩ *B|*/(*|A|* +*|B|*) and describes the relative overlap of segmentation volumes A and B. The DSC values range from 0 to 1 with a score of 0 indicating no overlap and 1 indicating perfect overlap. In addition, the HD was used to assess the amount of gross error between contours. The 3D HD is the maximum distance of a point in one contour to the nearest point of the other contour: *h*(*A,B*) = *max*_*a*∈*A*_[*min*_*b*∈*B*_[*d*(*a,b*)]], in which *a* and *b* are points in sets A and B, respectively, and *d(a,b)* is the Euclidean metric between these points [11]. Each manual contour, corrected autocontour, and autocontour was compared to the best manual contour by using DSCs and HDs. Sensitivity analysis was then conducted, comparing each contour with the second-best manual contour instead of the first-best manual contour, by using DSCs and HDs. We assessed whether the results achieved with the second-best manual contour were consistent with the primary results achieved with the first-best manual contour. To assess the time-saving effect, recorded videos on contouring were centrally reviewed and the contouring times for all nine OARs and for each OAR were measured. The times for manual contouring and correcting the autocontours were compared. To evaluate user satisfaction, questionnaires were sent to 11 experts to estimate the efficacy and feasibility of using the proposed ACS: question 1 was “How was the accuracy of the autocontours?”; question 2 was “How much did the autocontours help in shortening the contouring time?”; and question 3 was “Do you want to use autocontours in future practice?”. The answers were given numerical values ranging from 0 (i.e., “worst”) to 10 (i.e., “best”).

### Statistical analyses

To determine the accuracy of OARs volumes, DSCs and HDs were compared between manual contours, corrected autocontours, and autocontours using the paired *t*-test. For group-wise comparisons, *P*-values were corrected with Bonferroni’s method to counter the problem of multiple comparison. Values of *P* < 0.05 were considered statistically significant. Statistical calculations were conducted using SPSS software (version 25; IBM, Armonk, NY) and GraphPad Prism Version 8 (GraphPad Software, San Diego, CA).

## Results

### Accuracy

We collected 110 manual contours, 110 corrected autocontours, and 10 autocontours of each type of OAR. When these contours were compared to the consensus ground truth contours, 100 DSCs and 100 HDs (i.e., pairs of the ground truth contour and each contour) were created for each type of OAR for the manual contours and corrected autocontours, and 10 DSCs and 10 HDs were created for the autocontours.

**Fig. 1:**
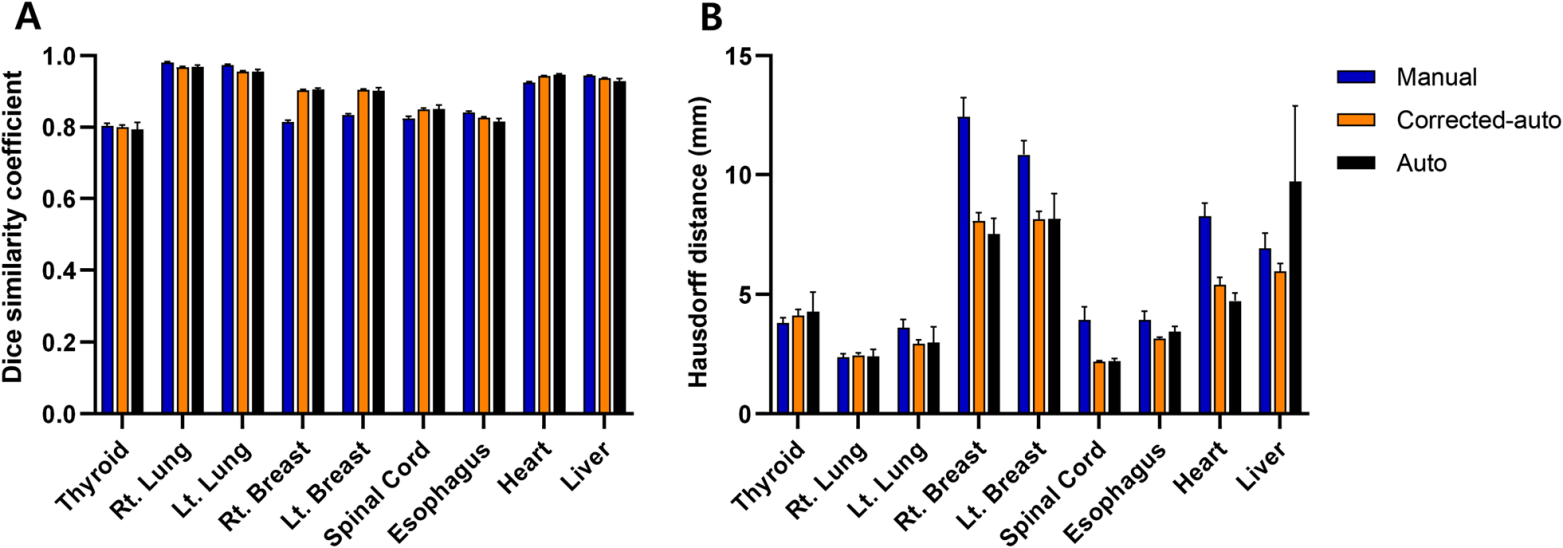
**A** Dice similarity coefficient and **B** Hausdorff distance values, based on the organs at risk. The manual contours, corrected autocontours, and autocontours are compared. Data are presented as the mean ± standard error

Table 1 and Fig. 1 show the mean DSCs and HDs of the manual contours, corrected autocontours, and autocontours for each OAR. In general, manual contours, corrected autocontours, and autocontours had a similar accuracy from the average DSCs (0.88 vs. 0.90 vs. 0.90). However, for breast contours, the DSCs were significantly higher in the corrected autocontours and autocontours than in the manual contours. The corrected autocontours of the breast had better DSCs than the manual contours by 0.09 (right breast) and 0.07 (left breast). In contrast, the absolute difference in the DSCs between the corrected autocontours and manual contours for other OARs was relatively small. For example, the absolute difference in DSCs between the two groups was 0.01 for the lungs, < 0.01 for the thyroid, 0.03 for the spinal cord, 0.01 for the esophagus, 0.02 for the heart, and < 0.01 for the liver.

**Table 1:** Summary of DSC and HD

		Contour			*P*-value*		
		(1) Manual (n = 100)	(2) Corrected auto (n = 100)	(3) Auto (n = 10)	(1) vs. (2)	(1) vs. (3)	(2) vs. (3)
DSC (mean ± SE)	Thyroid	0.80 ± 0.01	0.80 ± 0.01	0.79 ± 0.02	.953	.042	.014
	Lung_right	0.98 ± 0.00	0.97 ± 0.00	0.97 ± 0.00	< .001	< .001	.262
	Lung_left	0.97 ± 0.00	0.96 ± 0.00	0.96 ± 0.01	< .001	< .001	.801
	Breast_right	0.81 ± 0.00	0.90 ± 0.00	0.91 ± 0.00	< .001	< .001	.596
	Breast_left	0.83 ± 0.00	0.90 ± 0.00	0.90 ± 0.01	< .001	< .001	.280
	Spinal cord	0.82 ± 0.01	0.85 ± 0.00	0.85 ± 0.01	.001	.001	.136
	Esophagus	0.84 ± 0.00	0.83 ± 0.00	0.82 ± 0.01	.001	< .001	< .001
	Heart	0.92 ± 0.00	0.94 ± 0.00	0.95 ± 0.00	< .001	< .001	.029
	Liver	0.94 ± 0.00	0.94 ± 0.00	0.93 ± 0.01	< .001	< .001	< .001
HD (mean ± SE)	Thyroid	3.82 ± 0.20	4.12 ± 0.26	4.28 ± 0.83	0.33	0.056	0.021
	Lung_right	2.37 ± 0.15	2.46 ± 0.09	2.42 ± 0.29	> .999	> .999	0.472
	Lung_left	3.61 ± 0.35	2.93 ± 0.18	2.99 ± 0.67	0.077	0.115	0.538
	Breast_right	12.44 ± 0.80	8.06 ± 0.35	7.54 ± 0.65	< .001	< .001	0.284
	Breast_left	10.85 ± 0.58	8.14 ± 0.33	8.15 ± 1.05	< .001	< .001	> .999
	Spinal cord	3.95 ± 0.54	2.20 ± 0.04	2.21 ± 0.11	0.005	0.005	0.475
	Esophagus	3.95 ± 0.36	3.16 ± 0.06	3.46 ± 0.21	0.1	0.574	< .001
	Heart	8.26 ± 0.55	5.42 ± 0.29	4.73 ± 0.33	< .001	< .001	0.034
	Liver	6.92 ± 0.64	5.95 ± 0.32	9.74 ± 3.16	0.335	0.085	0.002

*Abbreviations:* DSC, Dice similarity coefficient; HD, Hausdorff distance; SE, standard error

^*^P-values were calculated using the paired *t*-test with Bonferroni correction

The HDs of breast and heart contours were significantly lower in the corrected autocontours and autocontours than in the manual contours. The HD of liver contours were significantly higher in the autocontours than in the corrected autocontours. The results of the sensitivity analyses were consistent with the original analyses for all OARs, excluding the thyroid and lungs (Additional file 1: Table S1 and Additional file 1: Figure S1).

To evaluate the performance of autocontouring alone, the average DSCs and HDs of all nine OARs were compared between manual contours and autocontours. In the manual contours, the average values of the DSCs of all OARs ranged from 0.870 to 0.903 (median, 0.881), depending on the expert, and the average values of HDs of all OARs ranged from 5.327 mm to 7.636 mm (median, 6.431 mm). Based on these DSCs, autocontours ranked second with a value of 0.896, after the manual contour’s value of 0.903. Based on these HDs, autocontours ranked first place with a value of 5.142 mm, followed by manual contours with a value 5.327 mm (Table 2).

**Table 2:** The DSC and HD values of all organs at risk (n = 9) of the experts’ manual contours and autocontours, listed from the best to the lowest performance

Rank	DSC	HD
	Average (standard error)	
1	0.903 (0.022)	5.142 (0.897)*
2	0.896 (0.020)*	5.327 (1.208)
3	0.887 (0.021)	5.477 (1.018)
4	0.886 (0.020)	5.615 (1.181)
5	0.882 (0.022)	5.780 (1.240)
6	0.881 (0.020)	6.431 (1.606)
7	0.881 (0.025)	6.447 (1.979)
8	0.880 (0.030)	6.461 (1.340)
9	0.877 (0.022)	6.501 (1.357)
10	0.874 (0.027)	6.724 (2.099)
11	0.870 (0.029)	7.636 (2.338)

*Abbreviations:* DSC, Dice similarity coefficient; HD, Hausdorff distance

^*^The value of an autocontour

### Interphysician variability

**Fig. 2:**
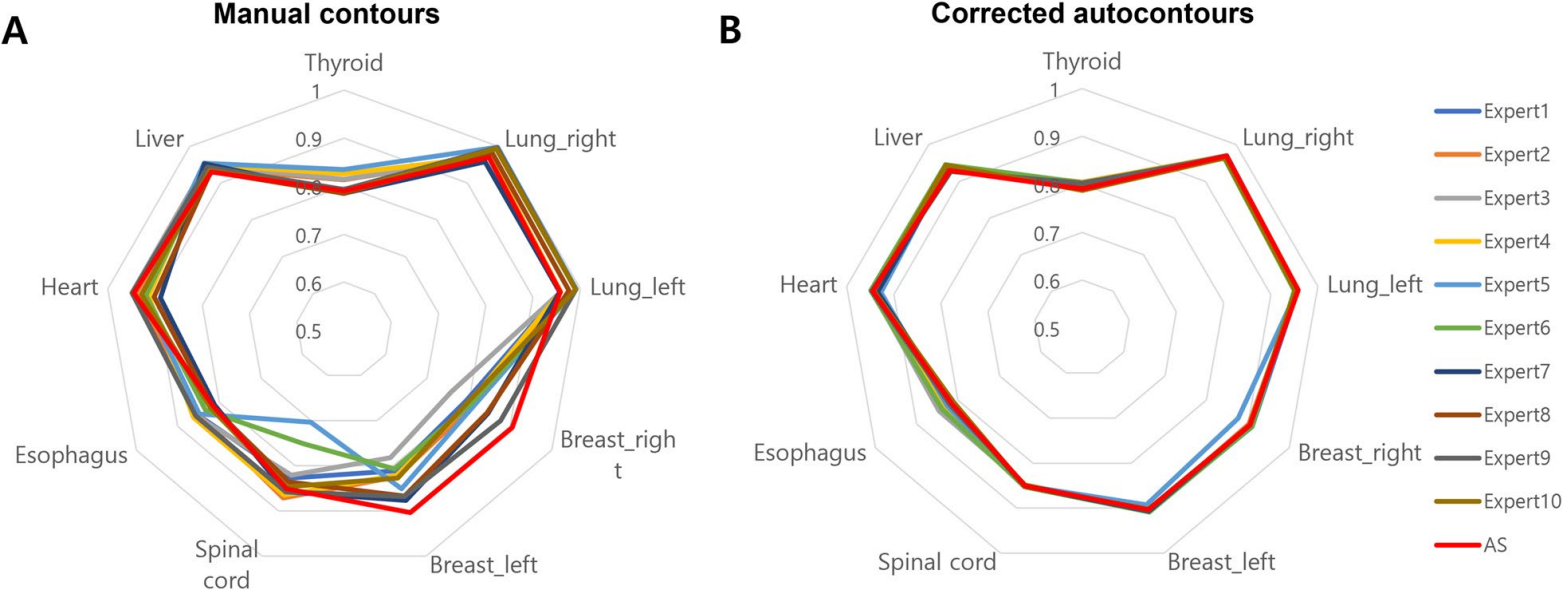
Radar graphs showing the mean Dice similarity coefficient value of each participant, based on the organ at risk. **A** Manual contours. **B** Corrected autocontours. The Dice similarity coefficient values of the corrected-autocontours are more homogeneous than those of the manual contours, which indicate reduced interphysician variability

The interphysician variations observed in the experts’ manual contours were reduced in the corrected autocontours. The mean DSCs of all OARs ranged from 0.87 to 0.90, based on the individuals’ manual contours, although the range was reduced to 0.89–0.90 in the individuals’ corrected autocontours. The mean HDs of all OARs ranged from 5.3 mm to 7.6 mm, based on the individuals’ manual contours, although the range was reduced to 4.3–5.8 mm in the individual’s corrected autocontours. Figure 2 shows the mean DSCs based on OARs. This figure shows that DSCs were more homogeneous in the corrected autocontours than in the manual contours, indicating reduced interphysician variability. The sensitivity analysis in Additional file 1: Figure S2 reveals results consistent to those of the original analyses.

**Fig. 3:**
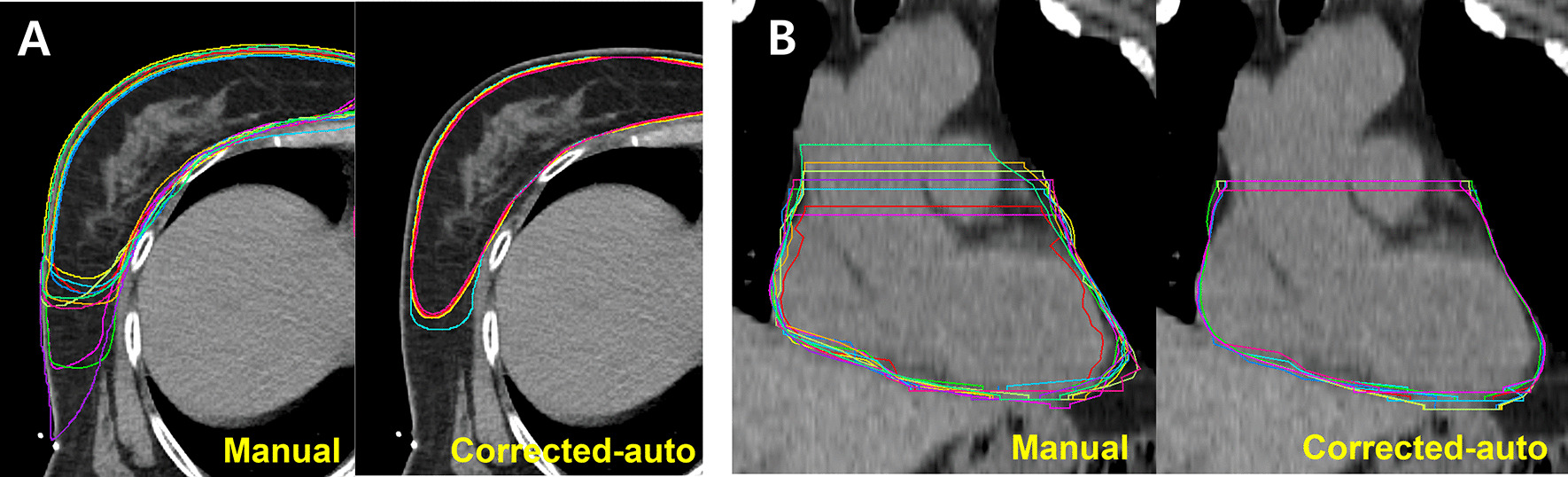
Examples of manual and corrected autocontours of all experts. **A** The breast contours show that interphysician variability in manual contours occurs mostly at the lateral and anterior borders of the breasts, and that this variability is reduced in corrected autocontours. **B** The heart contours show that interphysician variability in manual contours occurs mostly for the superior borders of the hearts, and that this variability is reduced in corrected autocontours

Examples of manual and corrected autocontours of the breast and heart are shown in Fig. 3. Of note, the interphysician variability of manual breast contours mostly occurred in the lateral and anterior borders of the breast, whereas this variability rarely occurred in the corrected autocontours. For heart contours, interphysician variability of the manual contours mostly occurred in the superior borders, whereas this variability rarely occurred in the corrected autocontours.

### Time-saving effect

**Fig. 4:**
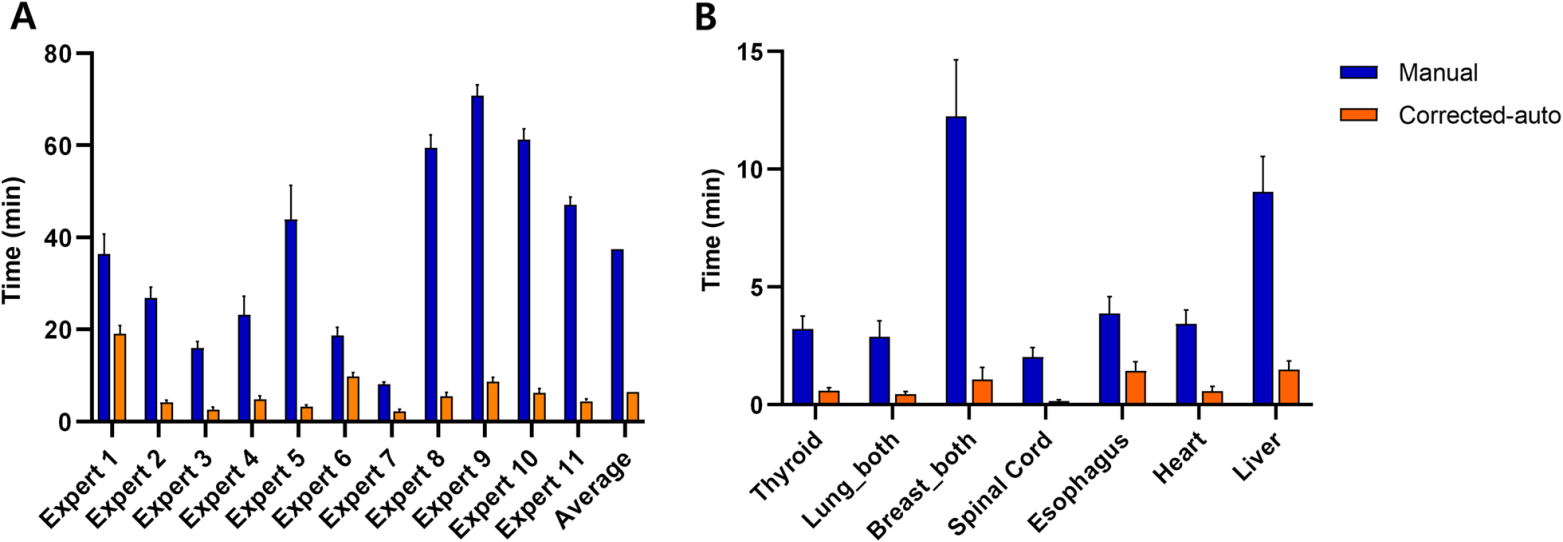
A comparison of the contouring time for manual contouring and corrected autocontouring. **A** The total contouring time of all nine organs at risk of each expert. **B** The contouring time of each organ at risk. Data are presented as the mean ± standard error

The mean (± standard error) contouring time for the nine OARs of each patient was 37.4 (± 5.9) min for the manual contours and 6.4 (± 1.4) min for corrected autocontours, which indicated a time reduction of 84% with the ACS (Fig. 4A and Additional file 1: Table S2). The process of obtaining autocontours was fully automated and took < 10 min, depending on the computer performance. When the mean time was measured, based on each OAR, breast and liver contouring was the longest step among the manual contours. The time was prominently reduced in the corrected autocontours [right breast: from 5.9 (± 1.2) min to 0.5 (± 0.3) min; left breast: from 6.3 (± 1.2) min to 0.6 (± 0.2) min; liver: from 9.0 (± 1.5) min to 1.5 (± 0.4) min] (Fig. 4B and Additional file 1: Tables S3 and S4).

### User satisfaction

The mean (± standard error) scores of questions regarding user satisfaction were as follows: question 1, 7.5 (± 0.3); question 2, 8.8 (± 0.3); and question 3, 9.2 (± 0.2).

## Discussion

Deep learning-based autosegmentation has been widely investigated in head and neck, thoracic, and genitourinary malignancies, although there is relatively less data regarding the use of deep learning-based autosegmentation in breast radiotherapy planning, for which conventional field-based and 2D radiotherapy techniques are commonplace. In this context, we previously developed a deep learning-based ACS for breast radiotherapy planning and reported its feasibility [7]. The clinical target volumes for the breasts and OARs were manually contoured in simulation CT scans and trained with a 3D U-Net-like CNN.

By using our deep learning-based ACS, we compared the performance between manual contours, corrected autocontours, and autocontours with multiple experts from two institutions. We showed that autocontours and corrected autocontours were closer to the ground truth than were manual contours. Furthermore, the accuracy of autocontours for breasts and OARs was as good as that of other deep learning-based ACSs developed by Men et al. [5] and Feng et al. [6]. The interphysician variation in manual contours was greatly reduced with the ACS. Moreover, the time spent contouring was substantially reduced with the ACS. Satisfaction was good among participants using the ACS.

Before the era of deep learning-based autosegmentation, atlas-based autosegmentation was actively studied in breast cancer and in other cancer sites. In a previous study [8], we compared the performance of deep learning-based autosegmentation with that of atlas-based autosegmentation in breast OARs and clinical target volumes. The proposed deep learning-based autosegmentation showed more consistent results and outperformed atlas-based autosegmentation in most structures. The next clinically relevant issue would be to address how the performance of the proposed deep-learning algorithm, which was trained by a single expert, would compare with that of a group of experts.

The novelty of the current study is that experts’ manual contours and autocontours were compared with the ground truth, which was determined by the other third-party experts. The average DSC and HD of autocontours ranked second and first place, respectively, in relation to the experts’ manual contours. This finding indicated that the autocontours have, at least, a similar performance as that of the experts’ manual contours. The good performance of autocontours may be because of the good quality of the training dataset for deep learning systems attached to the contouring guidelines [4]. In addition, possible human errors in manual contouring may be caused by fatigue from repetitive work. Another strength of this study is the integration of experts with diverse clinical backgrounds and participation of different institutions.

We observed substantial interphysician variability between the experts’ manual contours. Substantial variability in the manual contouring of the targets and OARs between institutions and observers was demonstrated in a Radiation Therapy Oncology Group (RTOG) multi-institutional and multiobserver study [12]. Such interphysician variability is an obstacle in accurately assessing the efficacy of radiotherapy and risk of long-term adverse effects. Incidental radiation exposure to the heart during breast radiation therapy increases the risk of heart disease with regard to the dose–response relationship between heart radiation dose and an acute coronary event [13, 14]. Moreover, radiation-related hypothyroidism [15], radiation pneumonitis [16], and secondary contralateral breast cancer [17] have been reported in patients with breast cancer. In addition, in clinical trials including radiotherapy, standardization of treatment is problematic because of the variability in delineating the target and OARs [18]. In the RTOG 0617 trial [19], a radiation dose-escalation trial of non-small cell lung cancer, an analysis using deep-learning segmented hearts revealed that the actual heart doses were higher than originally reported owing to inconsistent and insufficient manual heart segmentation. Our results demonstrated that the ACS could solve this issue. For example, the lateral border of the breast had the largest variation among the experts’ manual contours in this study. The most widely used RTOG guidelines [20] and ESTRO guidelines [21] define the lateral border of the breast as a clinically palpable breast or lateral breast fold; therefore, clearly defining it on a CT image is difficult. The ACS can aid in standardizing delineation when the definition of the boundary of an organ is ambiguous.

For the breast and heart contours, the autocontours and corrected-autocontours had a significantly better accuracy than did the manual contours. By contrast, the HDs of liver contours were high in the autocontours and reduced in the corrected autocontours to a value similar to that of manual contours, suggesting that manual adjustment was necessary. Therefore, the detailed performance of ACS appears to vary depending on the OAR.

The manual adjustment of autocontouring had an average time reduction of 84%, compared with manual contouring. This was most remarkable in breast and liver contouring, which required the most time. When adjusting the autocontour, the average time taken for each organ was < 1 min, indicating that only minimal or no correction was needed. In addition, the participants responded that ACS helped to shorten the time spent contouring and that they would like to use it in the future. According to Shanafelt et al. [22], symptoms of burnout have been reported in > 50% of practicing physicians, and this affliction is largely driven by work-related stressors [23]. Therefore, efforts are needed to reduce the workload of physicians. The ACS can be used for this.

This study has a limitation. It is difficult to determine the ultimate ground truth. Although the ground truth was determined by a separate qualified group of attending physicians, other experts may not agree with the ground truth. Thus, to clarify the results, we conducted the same analysis by using the second-best contours instead of the ground truth contours for the sensitivity analysis. The results were generally consistent with the original analyses for all OARs, excluding the thyroid and lungs.

## Conclusions

The ACS can overcome several weaknesses of manual contouring, such as labor intensity, time consumption, and interphysician variation. To expand the frame of this study, we are conducting a multicenter study in the Korean Radiation Oncology Group to examine the effectiveness of the ACS in the breast target. In the future, delineating OARs and accurately assessing the toxicity risk by the irradiated dose of each organ will become more important in breast cancer radiotherapy as treatments become more sophisticated. Adopting the ACS in breast cancer radiotherapy could be helpful in this regard.

## Supplementary Information

**Additional file 1.** **Table S1**. Summary of DSC and HD for sensitivity analyses; **Table S2**. Total contouring time for all organs at risk of each patient; **Table S3**. Time for manual contouring, according to each organ at risk; **Table S4**. Time for correcting autocontours, according to each organ at risk; **Figure S1**. (A) Dice similarity coefficient and (B) Hausdorff distance values, based on the organ at risk. Manual contours, corrected autocontours, and autocontours are compared. For the sensitivity analyses, contouring metrics were obtained by comparing each contour with the secondbest contour; **Figure S2**. Radar graphs showing the mean Dice similarity coefficient value of each participant, based on the organ. (A) Manual contours. (B) Corrected autocontours. The Dice similarity coefficient values of the corrected autocontours were more homogeneous than those of the manual contours, which indicate reduced interphysician variability. For sensitivity analyses, contouring metrics were obtained by comparing each contour with the second-best contour.

## Data Availability

The datasets used and/or analyzed during the current study available from the corresponding author on reasonable request.

## Abbreviations

2D: Two-dimensional
3D: Three-dimensional
ACS: Autocontouring system
CT: Computed tomography
DSC: Dice similarity coefficient
HD: Hausdorff distance
OAR: Organ at risk
RTOG: Radiation Therapy Oncology Group
