# Supplementary material for: Evaluation of deep learning-based autosegmentation in breast cancer radiotherapy
Source: Radiat Oncol. 2021 Oct 14;16:203. doi: 10.1186/s13014-021-01923-1 (PMC8518257; doi:10.1186/s13014-021-01923-1)
Supplement: Supplementary file 1 — Additional file 1. Table S1. Summary of DSC and HD for sensitivity analyses; Table S2. Total contouring time for all organs at risk of each patient; Table S3. Time for manual contouring, according to each organ at risk; Table S4. Time for correcting autocontours, according to each organ at risk; Figure S1. (A) Dice similarity coefficient and (B) Hausdorff distance values, based on the organ at risk. Manual contours, corrected autocontours, and autocontours are compared. For the sensitivity analyses, contouring metrics were obtained by comparing each contour with the secondbest contour; Figure S2. Radar graphs showing the mean Dice similarity coefficient value of each participant, based on the organ. (A) Manual contours. (B) Corrected autocontours. The Dice similarity coefficient values of the corrected autocontours were more homogeneous than those of the manual contours, which indicate reduced interphysician variability. For sensitivity analyses, contouring metrics were obtained by comparing each contour with the second-best contour. [file 13014_2021_1923_MOESM1_ESM.docx]

Table S1. Summary of DSC and HD for sensitivity analyses†

|  |  | Contour | | |  | *P*-value* | | |
| --- | --- | --- | --- | --- | --- | --- | --- | --- |
|  |  | (1) Manual  (n=100) | (2) Corrected-auto  (n=100) | (3) Auto  (n=10) |  | (1) vs. (2) | (1) vs. (3) | (2) vs. (3) |
| DSC (mean±SE) | Thyroid | 0.78±0.01 | 0.77±0.01 | 0.77±0.02 |  | 0.942 | 1.031 | >.999 |
|  | Lung_right | 0.96±0.00 | 0.97±0.00 | 0.96±0.00 |  | 0.099 | 0.214 | 0.336 |
|  | Lung_left | 0.96±0.00 | 0.96±0.00 | 0.96±0.01 |  | >.999 | 0.441 | 0.04 |
|  | Breast_right | 0.83±0.00 | 0.87±0.00 | 0.87±0.01 |  | <.001 | <.001 | 0.355 |
|  | Breast_left | 0.85±0.00 | 0.88±0.00 | 0.88±0.01 |  | <.001 | <.001 | 0.971 |
|  | Spinal cord | 0.85±0.01 | 0.89±0.00 | 0.89±0.01 |  | <.001 | <.001 | >.999 |
|  | Esophagus | 0.80±0.00 | 0.75±0.00 | 0.74±0.01 |  | <.001 | <.001 | <.001 |
|  | Heart | 0.89±0.00 | 0.90±0.00 | 0.90±0.01 |  | 0.007 | 0.001 | 0.066 |
|  | Liver | 0.95±0.00 | 0.94±0.00 | 0.93±0.01 |  | <.001 | <.001 | <.001 |
| HD (mean±SE) | Thyroid | 4.16±0.19 | 3.99±0.13 | 3.98±0.34 |  | 0.956 | 0.962 | >.999 |
|  | Lung_right | 4.04±0.20 | 4.12±0.16 | 4.29±0.52 |  | >.999 | 0.421 | 0.003 |
|  | Lung_left | 3.45±0.20 | 3.54±0.20 | 3.84±0.79 |  | >.999 | 0.045 | 0.006 |
|  | Breast_right | 14.28±0.66 | 11.7±0.49 | 11.87±1.62 |  | 0.001 | 0.005 | >.999 |
|  | Breast_left | 11.51±0.40 | 9.27±0.35 | 9.50±1.22 |  | <.001 | 0.002 | 0.524 |
|  | Spinal cord | 2.15±0.05 | 1.71±0.03 | 1.73±0.10 |  | <.001 | <.001 | 0.134 |
|  | Esophagus | 3.29±0.10 | 4.40±0.13 | 4.60±0.24 |  | <.001 | <.001 | 0.411 |
|  | Heart | 12.90±0.64 | 12.99±0.56 | 12.21±1.62 |  | >.999 | 0.99 | 0.015 |
|  | Liver | 6.11±0.32 | 6.83±0.35 | 10.37±2.70 |  | 0.258 | <.001 | 0.001 |

*Abbreviations:* DSC, Dice similarity coefficient; HD, Hausdorff distance; SE, standard error

* *P*-values were calculated using the paired *t*-test with Bonferroni correction.

† Sensitivity analysis was then conducted by comparing each contour with the second-best manual contour instead of the first-best manual contour

Table S2. Total contouring time for all organs at risk of each patient

|  | Manual (min) | Corrected-auto (min) | Difference* (min) |
| --- | --- | --- | --- |
| Expert 1 | 36.4 | 19.1 | 17.3 |
| Expert 2 | 26.9 | 4.2 | 22.6 |
| Expert 3 | 16.0 | 2.6 | 13.4 |
| Expert 4 | 23.2 | 4.8 | 18.4 |
| Expert 5 | 43.9 | 3.2 | 40.7 |
| Expert 6 | 18.7 | 9.8 | 8.9 |
| Expert 7 | 8.1 | 2.2 | 5.9 |
| Expert 8 | 59.4 | 5.5 | 53.9 |
| Expert 9 | 70.8 | 8.7 | 62.1 |
| Expert 10 | 61.2 | 6.2 | 55.1 |
| Expert 11 | 47.1 | 4.4 | 42.7 |
| Mean | 37.4 | 6.4 | 31.0 |
| SE | 5.9 | 1.4 | 5.9 |

*Abbreviations:* SE, standard error

* Time for correcting the autocontours minus the time for manual contouring

Table S3. Time for manual contouring, according to each organ at risk

|  | Thyroid (min) | Lung_right (min) | Lung_left (min) | Breast_right (min) | Breast_left (min) | Spinal Cord (min) | Esophagus (min) | Heart (min) | Liver (min) |
| --- | --- | --- | --- | --- | --- | --- | --- | --- | --- |
| Expert 1 | 4.3 | 1.3 | 1.5 | 5.1 | 6.1 | 1.2 | 4.4 | 3.7 | 8.0 |
| Expert 2 | 1.7 | 1.4 | 0.6 | 5.8 | 6.2 | 1.2 | 1.6 | 1.7 | 5.9 |
| Expert 3 | 1.7 | 0.4 | 0.8 | 1.8 | 1.7 | 0.9 | 2.3 | 1.6 | 3.6 |
| Expert 4 | 2.0 | 0.5 | 0.6 | 1.8 | 2.4 | 1.3 | 2.3 | 1.6 | 7.2 |
| Expert 5 | 2.4 | 4.9 | 3.9 | 6.1 | 6.7 | 3.2 | 4.1 | 4.3 | 7.5 |
| Expert 6 | 1.8 | 0.9 | 0.9 | 2.6 | 2.3 | 0.8 | 2.1 | 1.7 | 5.7 |
| Expert 7 | 1.1 | 0.6 | 0.4 | 0.8 | 0.8 | 0.7 | 0.5 | 1.1 | 1.6 |
| Expert 8 | 4.0 | 3.3 | 2.2 | 11.2 | 10.7 | 4.0 | 6.7 | 6.2 | 11.1 |
| Expert 9 | 5.4 | 1.9 | 1.3 | 12.8 | 14.8 | 4.3 | 7.9 | 6.2 | 16.1 |
| Expert 10 | 7.0 | 1.7 | 1.0 | 8.7 | 8.9 | 2.8 | 7.3 | 6.2 | 17.6 |
| Expert 11 | 4.1 | 1.2 | 0.6 | 8.7 | 8.7 | 2.0 | 3.4 | 3.3 | 15.1 |
| Mean | 3.2 | 1.6 | 1.2 | 5.9 | 6.3 | 2.0 | 3.9 | 3.4 | 9.0 |
| SE | 0.5 | 0.4 | 0.3 | 1.2 | 1.2 | 0.4 | 0.7 | 0.6 | 1.5 |

*Abbreviations:* SE, standard error

Table S4. Time for correcting autocontours, according to each organ at risk

|  | Thyroid (min) | Lung_right (min) | Lung_left (min) | Breast_right (min) | Breast_left (min) | Spinal Cord (min) | Esophagus (min) | Heart (min) | Liver (min) |
| --- | --- | --- | --- | --- | --- | --- | --- | --- | --- |
| Expert 1 | 1.4 | 0.2 | 1.2 | 3.9 | 2.4 | 0.6 | 2.5 | 2.4 | 3.5 |
| Expert 2 | 0.1 | 0.1 | 0.1 | 0.4 | 0.5 | 0.0 | 0.2 | 0.4 | 1.2 |
| Expert 3 | 0.1 | 0.1 | 0.1 | 0.0 | 0.2 | 0.0 | 0.8 | 0.0 | 0.6 |
| Expert 4 | 0.7 | 0.1 | 0.1 | 0.1 | 0.2 | 0.2 | 0.6 | 0.4 | 1.0 |
| Expert 5 | 0.1 | 0.5 | 0.2 | 0.1 | 0.1 | 0.3 | 0.1 | 0.0 | 0.4 |
| Expert 6 | 1.1 | 0.1 | 0.0 | 0.1 | 0.9 | 0.1 | 1.5 | 1.2 | 4.2 |
| Expert 7 | 0.1 | 0.2 | 0.1 | 0.0 | 0.0 | 0.1 | 0.0 | 0.3 | 0.7 |
| Expert 8 | 0.7 | 0.1 | 0.2 | 0.6 | 1.1 | 0.2 | 1.8 | 0.2 | 0.6 |
| Expert 9 | 1.1 | 0.7 | 0.3 | 0.2 | 0.6 | 0.3 | 3.6 | 1.0 | 1.0 |
| Expert 10 | 0.8 | 0.1 | 0.1 | 0.0 | 0.1 | 0.0 | 3.7 | 0.1 | 1.4 |
| Expert 11 | 0.5 | 0.2 | 0.2 | 0.0 | 0.1 | 0.0 | 1.1 | 0.4 | 1.9 |
| Mean | 0.6 | 0.2 | 0.2 | 0.5 | 0.6 | 0.2 | 1.5 | 0.6 | 1.5 |
| SE | 0.1 | 0.1 | 0.1 | 0.3 | 0.2 | 0.1 | 0.4 | 0.2 | 0.4 |

*Abbreviations:* SE, standard error

**Figure S1.** (A) Dice similarity coefficient and (B) Hausdorff distance values, based on the organ at risk. Manual contours, corrected autocontours, and autocontours are compared. For the sensitivity analyses, contouring metrics were obtained by comparing each contour with the second-best contour. Data are presented as the mean ± standard error.

Lt, left; Rt, right

**Figure S2.** Radar graphs showing the mean Dice similarity coefficient value of each participant, based on the organ. (A) Manual contours. (B) Corrected autocontours. The Dice similarity coefficient values of the corrected autocontours were more homogeneous than those of the manual contours, which indicate reduced interphysician variability. For sensitivity analyses, contouring metrics were obtained by comparing each contour with the second-best contour.

Lt, left; Rt, right
